# Rapid method for determination of DNA repair capacity in human peripheral blood lymphocytes amongst smokers

**DOI:** 10.1186/1471-2407-10-439

**Published:** 2010-08-18

**Authors:** Randa A El-Zein, Claudia M Monroy, Andrea Cortes, Margaret R Spitz, Anthony Greisinger, Carol J Etzel

**Affiliations:** 1 The University of Texas M. D. Anderson Cancer Center, Department of Epidemiology, Houston, Texas, USA; 2 Kelsey Research Foundation, Houston, Texas, USA

## Abstract

**Background:**

DNA repair capacity is an important determinant of susceptibility to cancer. The hOGG1 enzyme is crucial for repairing the 8-oxoguanine lesion that occurs either as a byproduct of oxidative metabolism or as a result of exogenous sources such as exposure to cigarette smoke. It has been previously reported that smokers with low hOGG1 activity had significantly higher risk of developing lung cancer as compared to smokers with high hOGG1 activity.

**Methods:**

In the current study we elucidate the association between plasma levels of 8-OHdG and the OGG1 repair capacity. We used the commercially available 8-OHdG ELISA (enzyme-linked immunosorbent assay), the Comet assay/FLARE hOGG1 (Fragment Length Analysis by Repair Enzymes) assay for quantification of the levels of 8-OHdG and measured the constitutive, induced and unrepaired residual damage, respectively. We compared the DNA repair capacity in peripheral blood lymphocytes following H_2_O_2 _exposure in 30 lung cancer patients, 30 non-, 30 former and 30 current smoker controls matched by age and gender.

**Results:**

Our results show that lung cancer cases and current smoker controls have similar levels of 8-OHdG lesions that are significantly higher compared to the non-smokers controls. However, lung cancer cases showed significantly poorer repair capacity compared to all controls tested, including the current smokers controls. After adjustment for age, gender and family history of smoking-related cancer using linear regression, we observed a 5-fold increase in risk of lung cancer associated with high levels of residual damage/reduced repair capacity. Reduced OGG1 activity could be expected to be a risk factor in other smoking-related cancers.

**Conclusion:**

Our study shows that the Comet/FLARE assay is a relatively rapid and useful method for determination of DNA repair capacity. Using this assay we could identify individuals with high levels of residual damage and hence poor repair capacity who would be good candidates for intensive follow-up and screening.

## Background

DNA repair is a ubiquitous defense mechanism that is critical to maintaining the integrity of the genome. Several prior studies have reported associations between reduced repair capacity and cancer development [1-3]. Base excision repair (BER) is the main pathway involved in the repair of oxidative DNA damage and recent studies have indicated a correlation between reduced BER capacity and oncogenesis [4,5]. The oxidative DNA damage induced by reactive oxygen species (ROS) may lead to single- or double-strand breaks, point and frame-shift mutations and larger-scale chromosome abnormalities [6]. ROS can be formed endogenously as a result of xenobiotic metabolism by cytochrome P450 enzymes, by redox cycling of hormone metabolites or exposure to other environmental factors [7].

Smoking is an inducer of oxidative stress which results in ROS-induced DNA damage [8] in the form of DNA adducts. This may be of particular importance in lung cancer where increased oxidative DNA damage coupled with reduced BER may play an important role in modifying the disease risk [9]. It is well know that only a small percentage of smokers develop lung cancer [10] suggesting that individual DNA repair capacity may play a significant role in the carcinogenic process.

The 8-oxoguanine DNA glycosylase (hOGG1) is one of the key DNA repair enzymes involved in the BER pathway in humans [11,12]. It recognizes the 8-oxoguanine modifications from both nuclear and mitochondrial DNA [13]. Among the more than 30 different products of modified DNA and RNA by oxidative damage [14], 8-Hydroxy-2'-deoxyguanosine (8-OHdG) is the most extensively studied induced lesion. 8-OHdG lesion causes G→T and A→C transversions [15] that have been reported as the sites of spontaneous oncogene expression and ultimately cancer manifestation [16,17]. Deletion of the hOGG1 gene was shown to be associated with accumulation of 8-OHdG lesion and increase in mutational risk [18]. OGG1 deficiency in yeast was shown to result in a spontaneous mutator phenotype [19]. Collins et al observed a lack of repair activity on a DNA substrate containing 8-OHdG protein extracts from homozygous OGG1 knockout mouse embryonic fibroblasts [20]. In contrast, mammalian cells over-expressing OGG1 repair 8-OHdG more rapidly after toxic insult with oxidants [21]. Increased levels of 8-OHdG has been associated with an increased risk of lung cancer among smokers [22]. In a recent study, Paz-Elizur et al evaluated the enzymatic OGG1 activity among smoking lung cancer cases and controls. The authors reported that the OGG1 activity was significantly decreased in lung cancer cases, and that the risk of developing lung cancer in smokers with low OGG1 activity was significantly higher compared to smokers with high OGG1 activity [23]. To date, the association between the levels of 8-OHdG and OGG1 repair capacity has not been determined.

In the current study we hypothesized that an inverse association exists between the levels of 8-OHdG and OGG1 activity. Furthermore, we hypothesized that lung cancer patients have higher levels of 8-OHdG reflecting suboptimal OGG1 repair capacity when compared to healthy controls. To test these hypotheses, we quantified the levels of 8-OHdG and measured ROS-induced DNA damage and repair in peripheral blood lymphocytes of lung cancer cases and controls. We used the commercially available 8-OHdG ELISA (enzyme-linked immunosorbent assay) to measure the levels of 8-OHdG in plasma and the Comet assay and hOGG1 FLARE (Fragment Length Analysis by Repair Enzymes) assay to measure the constitutive, induced and unrepaired residual damage by measuring DNA strand breaks and abasic sites. The Comet assay is used to detect DNA damage (including single-strand breaks, double-strand breaks and alkali-labile sites) [24,25] as well as to determine interindividual variation in DNA repair capacity through the measurement of residual DNA damage after exposure to mutagens [26,27]. The hOGG1 FLARE is a modified Comet assay that employs 8-oxoguanine DNA glycosylase to recognize and nick the DNA specifically at the 8-OHdG lesion thus resulting in strand breaks [28]. Cells not exposed to hOGG1 serve as a baseline for naturally occurring DNA breaks; while cells exposed to hOGG1 contain both naturally occurring breaks in DNA, as well as breaks caused by hOGG1 at places containing unrepaired 8-OHdG adducts [29]. Our results show that lung cancer cases and current smoker controls had higher levels of 8-OHdG compared to the former and non-smokers controls. Similarly, our results demonstrated that baseline DNA damage is comparable between lung cancer cases and current smoker controls but is significantly higher than that observed among former smokers and non-smokers controls. Further investigation revealed that lung cancer cases showed significantly poorer repair capacity than that observed in current smoker controls suggesting impairment of adduct removal. In conclusion, our study shows that the Comet/FLARE assay is a relatively rapid and useful method for determination of DNA repair capacity and that adduct removal by OGG1 is a mechanism that might be significantly impaired among smokers who develop lung cancer. Using this assay we could potentially identify individuals with high levels of residual damage and hence poor repair capacity. Identification of the small percentage of susceptible smokers with poor repair would allow for better prevention interventions.

## Methods

### Study population

A total of 120 participants were included in the study. Thirty smoker lung cancer patients were recruited from The University of Texas M. D. Anderson Cancer Center as part of an ongoing lung cancer case-control study. The patients had newly diagnosed, histologically confirmed lung cancer with no prior history of treatment (radiation or chemotherapy) upon enrollment in the study. Controls (n = 90) were matched to cases on age (+/-5 years) and gender and were recruited from the Kelsey-Seybold Clinics in Houston, Texas. The control participants consisted of 30 non-smokers, 30 former smokers and 30 current smokers with no previous history of cancer. After informed consent was obtained, a personal interview was conducted and information regarding socio-demographics, smoking history, alcohol consumption, occupational exposures, diet and family history of cancer was collected. There were no age and gender restrictions for study eligibility. The institutional review boards at both M. D. Anderson Cancer Center and Kelsey-Seybold Clinic approved this study. After providing informed consent, 10-mL blood was collected from all study participants.

### Peripheral blood lymphocyte isolation

Peripheral blood and lymphocytes were isolated using standard Ficoll-Histopaque method. Briefly, 10 ml of whole blood from each subject was drawn into heparinized tubes, layered over 10 ml of Histopaque -1077 (Sigma Aldrich Co., St. Louis, MO) at room temperature and centrifuged at 1500 rpm for 30 min. The mononuclear cells were removed from the interphase, washed twice with phosphate-buffered saline pH 7.2 and centrifuged at 1500 rpm for 10 min. Cells were suspended in 1 ml of RPMI 1640 medium and the cell membrane integrity was determined by Trypan Blue solution 0.4% and adjusted to a concentration of 1 × 10^6 ^cells/ml. Duplicate lymphocyte cultures were prepared for each study subject. Lymphocytes were then resuspended in RPMI 1640 medium supplemented with 1% L-glutamine, 100 U/ml of penicillin and of streptomycin (Gibco Invitrogen Corp., Grand Island, NY), 10% fetal calf serum, 2% PHA (Murex Biotech Ltd., Dartford, England, UK) and cultured in 24-well microplates for 24 h at 37°C in 5% CO_2 _prior to treatment.

### Establishment of hydrogen peroxide (H_2_O_2_) treatment conditions

We used H_2_O_2 _as the ROS-inducing agent in vitro. In order to establish the optimal conditions for exposure and repair kinetics, peripheral blood lymphocytes were isolated from 20 healthy controls. A dose-response curve was generated using in vitro treatment of lymphocytes with H_2_O_2 _(at a range of concentrations between 20-100 μM) for 15 min. Results from the dose response curve indicated that the 60 μM concentration, lead to a cell viability >85% and DNA damage that was significantly higher than the negative control (P < 0.001) (Additional file 1, Figure S1A).

The cells concentration was adjusted to 3 × 10^4 ^cells/ml and suspended in RPMI 1640 medium without FBS, supplemented with 2 mM L-glutamine, 100 U/ml of penicillin/streptomycin and 20% Alamar Blue cell assay ™(BioSource International Inc., Camarillo, CA, USA) to determine metabolic activity/cell viability of the cells. The cell viability was measured for each time of treatment with a spectrophotometer (Tecan, Genios Pro) at 535 nm excitation and 590 nm emission. Using Pearson's correlation we estimated the correlation between the most common parameters reported in the literature to determine damage by the Comet assay namely: the tail extent moment (TEM) and the tail length (TL). Our results indicated a pair-wise correlation of R^2 ^= 0.94-0.96 (P < 0.001) and therefore in the following sections we reported our findings using the TEM only.

### Optimization of recovery time for H_2_O_2_-induced damage/repair

A normal lymphoblastoid cell line (AH, [cat#GM14520], Coriell Cell Repositories, Camden, NJ, USA) was used to determine the optimal incubation time for recovery of in vitro H_2_O_2_-induced damage. Significant DNA damage TEM was observed after 15 min H_2_O_2_-treatment as compared to the untreated cells (mean + SD = 0.89 + 0.18 versus 0.47 + 0.07 respectively, P = 0.002). After 1 h incubation at 37°C to allow the cells to recover, a significant reduction in TEM was observed as compared to the H_2_O_2_-treated cells (mean ± SD = 0.65 ± 0.11 versus 0.89 ± 0.18 respectively, P = 0.007); however the damage was still significantly higher than that observed in the untreated cells (mean + SD = 0.65 + 0.11 versus 0.47 + 0.07 respectively, P = 0.01). Incubation of the cells for 2 h at 37°C showed a further reduction in the level of measured TEM when compared to the 1 h incubation (mean ± SD = 0.51 ± 0.05 versus 0.65 ± 0.11, respectively); however these differences were not statistically significant and we therefore decided to use the one-hour incubation in our subsequent experiments in order to accommodate the further experimental procedures that included a further incubation with the hOGG1 enzyme followed by denaturation and electrophoresis (Additional file 1, Figure S1B).

### Measurement of 8-OHdG levels

Baseline serum levels of 8-OHdG was measured using the commercially available 8-OHdG ELISA Kit (BIOXYTECH^® ^OXIS International Inc., Foster City, CA, USA) following manufacturer's recommendations. This kit is a competitive ELISA for quantitative measurement of 8-OHdG in tissue, serum or plasma resulting from oxidative damage to DNA.

### Comet/FLARE^® ^assay

Collins et al [20] first introduced this technique to study BER pathway. The principal of this assay depends on incubating cell extracts with a substrate of DNA containing specific lesions. Incubation of the substrate DNA with the specific enzymes allows for the uncovering of specific damaged bases. Treating lysed, immobilized cells with a DNA glycosylase, converts the base to an alkali sensitive site, followed by DNA unwinding that allows additional damage to be recognized. This leads to the introduction of breaks that are measured using the single-cell gel electrophoresis (Comet) assay. On the basis of the initial results, the lymphocytes were treated with a concentration of 60 μM H_2_O_2 _in a total volume of 200 μM RPMI at 4°C for 15 min. Cells were then incubated at 37°C for 1 h to allow the cells to recover. The cell suspension was then aspirated from the wells and washed with PBS pH 7.2. Cells were then embedded in a layer of low melting point agarose (LMA) at 37°C prepared in PBS and the mixtures were immediately transferred to the FLARE^®^-slides (Trevigen Inc.). Duplicate slides were prepared from each well. Slides were immersed in lysis solution (2.5 M NaCl, 100 mM EDTA pH 10, 10 mM Tris base, 1% sodium lauryl sarcosinate, 1% Triton X-100 and 1% DMSO) (Trevigen Inc.) for 1 h at 4°C. The slides were washed in FLARE buffer (250 mM HEPES-KOH pH 7.4, 2.5 M KCl and 250 mM EDTA) at room temperature three times over a 15 min period. 100 μl of hOGG1 (1:500) was then added to each sample and incubated at 37°C for 40 min (following manufacturer's recommendations) to allow for further cell recovery. The DNA of the nuclei in the agarose gels was denatured in electrophoresis buffer pH 12.1 (3 M NaCl, 500 mM EDTA) for 30 min at 4°C. Electrophoresis was performed in alkaline solution pH 13 applying 300 mA, 25 V for 30 min at 4°C. The slides were then placed in cold methanol for 20 min, dried and stored in a slide box at room temperature. At time of analysis, the slides were hydrated in cold water at 4°C for 20 min and stained with ethidium bromide solution (2 μg/mL). Slides were coded in order to blind the treatment conditions from the scorer and 100 random cells/sample were analyzed (50 cells/duplicate slide) using an epi-fluorescence microscope (Nikon) equipped with Comet IV^® ^software (Perceptive Imaging, Haverhill, Suffolk, UK). The mean values of the TEM in each cell were computed for each experimental group.

### Statistical analysis

Descriptive statistics were used to characterize the cases and controls. Analysis of variance (ANOVA) was used to compare age and other continuous variables and the Chi-square test was used to compare the categorical variables. For the FLARE assay, the Percent Residual DNA Damage (PRD) was calculated using the following equation:

$PRD=\frac{X_{H_{2}O_{2}+OGG}-X_{H_{2}O_{2}}}{X_{H_{2}O_{2}}}\times \text{100}$. Where *XH_2_O_2 _*is the mean TEM values for each study subject after treatment with H_2_O_2_, while *X_H_2_O_2 _+ OGG _*is the mean TEM values for each study subject after H_2_O_2 _treatment and further incubation with OGG1 enzyme for removal of additional residual damage. Linear regression analysis was conducted to adjust the TEM H_2_O_2 _+ OGG1 by TEM H_2_O_2_. The residual values from the regression defined as Residual Damage Linear Regression (RDLR) among the cases and controls were computed using ANOVA. RDLR was dichotomized using the 75^th ^percentile of the controls and estimates of lung cancer risk (OR and 95% CL) for RDLR adjusted for age, sex and family history of a smoking related cancer were determined.

## Results

### Demographics and the study population

The demographic characteristics of the 30 cases and 90 controls are summarized in Table 1. No significant differences were observed between cases and the controls did not differ significantly in terms of age, gender or ethnicity. Fifty-two percent of the patients self-reported a family history of smoking-related cancer, compared with 40% of the current, 59% of the former and 50% of the non-smoker controls (P = 0.07). Cases had on an average smoked cigarettes for 44.23 years, compared with 42.70 and 35.37 years for current and former smoker controls (P > 0.05 and P < 0.001, respectively); however both cases and controls (current and former groups) smoked about the same number of cigarettes per day (mean number of cigarettes per day ± SE for cases = 29.2 ± 2.5 and 24.4 ± 1.75 and 35.0 ± 3.3 for current and former smokers respectively, P = 0.10).

**Table 1:** Comparison of demographic and clinical factors among cases and controls

	Cases	Controls			
		Current Smokers	Former Smokers	Non- Smokers	P-value
	N = 30	N = 30	N = 30	N = 30	
Age (mean ± SE)	62.7 ± 0.57	63.5 ± 0.84	61.9 ± 0.69	58.6 ± 0.98	0.27
Gender: N (%)					
Male	15(50.0)	20(66.7)	21(70.0)	20(66.7)	
Female	15(50.0)	10(33.3)	9(30.0	10(33.3)	0.58
Ethnicity					
Anglo	30(100.0)	29(96.7)	25(83.3)	30(100.0)	
Non-Anglo	0(0.0)	1(3.3)	5(16.7)	0(0.0)	0.24
No. of Cigarettes/day Mean ± SE	29.2 ± 2.5	24.4 ± 1.75	35.0 ± 3.3	0.0 ± 0.0	<0.001
					
Years smoked Mean ± SE:	44.23 ± 1.39	42.70 ± 1.46	35.37 ± 1.58	0.0 ± 0.0	<0.001
Family History Cancer					
N (%)					
No	14(48.3)	15(60.0)	12(41.4)	15(50.0)	
Yes	15(51.7)	10(40.0)	17(58.6)	15(50.0)	0.07

P-values from Chi-square test for association (categorical) or ANOVA (continuous)

### Measurement of 8-OHdG levels at baseline by ELISA

Among controls, the current and former smoker controls showed significantly higher levels as compared to non-smoker controls (mean ± SE, 1.99 + 0.55 ng/ml, 1.68 + 0.99 ng/ml versus 0.83 + 0.62 respectively, P < 0.001). There was no significant difference between the level of 8-OHdG among the former and current smokers controls (P = 0.28). Lung cancer cases had over 2-fold higher levels of 8-OHdG than the non-smoker controls (mean ± SE, 2.09 + 0.91 ng/ml versus 0.83 + 0.62, P < 0.001); however the difference was not significant when compared to former and current smoker controls (P = 0.88 and P = 0.33, respectively).

### Measurement of DNA damage at baseline by Comet assay

Table 2 shows the extent of baseline DNA damage measured as TEM. Among controls, the non- and former smokers had significantly lower DNA damage than the current smoker controls (mean ± SE = 0.26 ± 0.11 and 0.23 ± 0.09 versus 0.54 ± 0.15, P < 0.001). There was no significant difference between the level of baseline DNA damage among the non- and former smokers controls (P = 0.95).

**Table 2:** Distribution of baseline and H_2_O_2_-induced DNA damage among cases and controls

	Controls			Cases	P-value
Tail Extent Moment Mean ± SE	Non- Smokers	Former Smokers	Current Smokers	Current Smokers	
Baseline DNA damage	0.26 ± 0.11	0.23 ± 0.09	0.54 ± 0.15	0.57 ± 0.12	<0.001
H_2_O_2_- induced DNA damage	3.2 ± 0.82	5.8 ± 0.31	10.2 ± 0.93	19.5 ± 0.41	<0.001

Comparing cases and controls, the baseline levels of DNA damage was significantly different between groups (0.57 ± 0.12 versus 0.35 ± 0.19, P < 0.001). However, when the controls were subdivided by smoking status, the levels of TEM were significantly different between lung cancer cases and non- and former smoker controls only (P < 0.001). There was no difference between the level of baseline DNA damage among the current smoker controls and the lung cancer cases (mean ± SE = 0.54 ± 0.15 versus 0.57 ± 0.12 respectively, P = 0.83).

### Measurement of H_2_O_2_-induced DNA damage by Comet assay

Level of H_2_O_2_-induced DNA damage after an hour incubation (to allow cellular intrinsic repair of damage) is shown in Table 2. The level of TEM in all three groups of controls was significantly different with mean + SD = 3.2 + 0.82; 5.8 + 0.31 and 10.2 + 0.93 for non- former and current smoker controls, respectively. Lung cancer cases had over 6-, 3-and 1-fold higher levels of induced DNA damage than the non-, former and current smoker controls, respectively. The lung cancer cases had the highest level of DNA damage followed by the current smokers (mean + SD = 19.5 + 0.41 versus 10.2 + 0.93, P < 0.001). The non-smokers exhibited the least damage (mean + SD = 3.2 + 0.82 versus 5.8 + 0.31, 10.2 + 0.93, P < 0.001, for former and current smoker controls, respectively).

### Measurement of DNA repair and residual capacity by FLARE assay

To elucidate the underlying mechanisms underlying the differences in response between the lung cancer cases and the current smoker controls, we used the FLARE assay to measure the repair capacity between the 2 groups. The TEM measured by the FLARE assay is the end result of the H_2_O_2_-induced damage as well as a manifestation of the removal of additional 8-oxoG lesions recognized by the hOGG1 enzyme. Thus the FLARE can be used to measure the residual damage that has not been removed during the initial one hour recovery after H_2_O_2 _treatment. Lung cancer cases had over 2-fold higher levels of DNA damage than the current smoker controls (mean ± SD = 44.6 ± 0.89 versus 18.4 ± 1.37, P < 0.001). The residual DNA damage among cases and current smoker controls are summarized in Table 3. Comparing cases and current smokers controls revealed significantly lower levels of residual DNA damage in the controls (P < 0.001), thus reflecting more efficient repair capacity compared to the cases.

**Table 3:** Distribution of DNA damage and percent residual DNA damage among cases and current smoker controls

Tail Extent Moment Mean ± SE	Cases	Controls	P-value	Adjusted OR	95% CL
	Current Smokers	Current Smokers			
H_2_O_2 _+ OGG1	44.6 ± 0.89	18.4 ± 1.37	<0.001		
Percent Residual Damage (PRD)	237.4 ± 8.7	180.8 ± 50.7	<0.001		
					
Residual Damage Logistic Regression (RDLR)					
≤75th N (%)	13(43.3)	41(75.9			
>75th N (%)	17(56.7)	13(24.1)			
	5.07	1.80-14.24			
		Referent		5.07	1.80-14.24

P-value from t-test; TEM RDLR dichotomized by 75^th ^percentile of the controls. Adjusted by age, sex and family history of a smoking-related cancer

**Figure 1:**
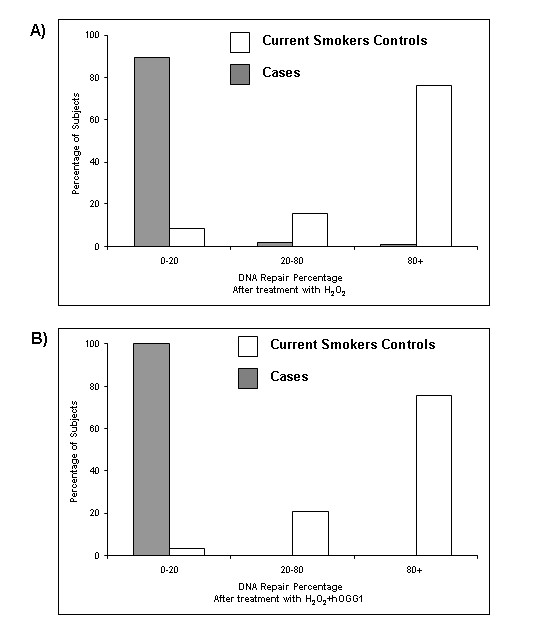
**Range of calculated DNA repair capacity measured as TEM among cases and current smoker controls**. A) Percentage of DNA repair capacity among current smoker controls and lung cancer cases, after treatment with H_2_O_2_. B) Percentage of DNA repair capacity among current smoker controls and lung cancer cases, after treatment with H_2_O_2 _+ hOGG1.

Figure 1 shows the range of calculated DNA repair capacity in response to H_2_O_2 _treatment (Figure 1A) and H_2_O_2 _+ hOGG1 (Figure 1B) among cases and current smokers controls using the 75^th ^percentile of the controls as the cutoff point. Minimal variation in repair was observed among the cases, with about 90% and 100% of the cases falling into the poor repair group (<20%) in response to H_2_O_2 _treatment alone and H_2_O_2 _+ hOGG1 treatments, respectively. Only 1% of the cases showed a repair capacity over 80%. In contrast, the current controls had a wide range of repair, with 76% of the control subjects having a repair capacity of >80% in response to the H_2_O_2 _treatment and H_2_O_2 _+ hOGG1 treatments. Table 2 shows the distribution of residual damage among cases and current controls and determination of risk for lung cancer. After adjustment for age, gender and family history of a smoking-related cancer using linear regression there was a 5-fold increase in risk of lung cancer associated with high levels of residual damage or reduced repair capacity (OR = 5.07; 97% CL = 1.80-14.24).

## Discussion

DNA damage generated by ROS produced as byproducts of cellular metabolism has been proposed as a key factor in mutagenesis and cancer process. Superoxide (O_2_^-^), hydrogen peroxide (H_2_O_2_), and the hydroxyl radical (OH^-^) are mutagens produced as byproducts of normal metabolism in mitochondria and by ionizing radiation [7]. ROS can induce single-strand breaks and several types of DNA base damage, including fragmented, ring-opened forms and oxidized aromatic derivatives. Among many repair pathways, the BER pathway is the most important cellular protection mechanism responding to oxidative DNA damage induced by ROS [5]. Therefore, increased oxidative DNA damage and reduced BER capacity may play an important role in lung carcinogenesis.

OGG1 is a critical component of the BER pathway required for the removal of 8-OHdG lesion from DNA exposed to ROS. The impaired repair activity of OGG1 protein was suggested to be a factor contributing to the high somatic mutation rate in human cells, since the accumulation of 8-OHdG cause mutations by mis-pairing with adenine during replication [11-14]. It has also been reported that lower enzymatic levels of OGG1 in peripheral lymphocytes correlated with an increased risk of lung cancer among smokers [23]. In our current study, we elucidate the association between plasma levels of 8-OHdG and the OGG1 repair capacity among lung cancer cases and controls.

Our results demonstrated that baseline DNA damage is similar among lung cancer cases and current smoker controls and is significantly higher than that observed among the former and non-smokers controls. These findings are consistent with other studies showing higher levels of constitutive DNA damage in the form of spontaneous chromosomal aberrations and elevated endogenous single-stranded breaks [30-32] among patients compared to controls. Results from the baseline serum levels of 8-OHdG followed a similar pattern to that of the Comet assay where lung cancer cases and current smoker controls had the highest levels of 8-OHdG as compared to the former smokers; however, these differences were not significant. Interestingly, the levels of 8-OHdG were significantly lower among non-smoker controls as compared to the lung cancer cases and current smoker controls. These findings are consistent with studies that have reported that smoking-induced DNA adducts persist for a long time and could still be detectable long after quitting [33,34]. Moreover, numerous reports have associated radicals with smoking-related oxidative stress and DNA damage [23,35,36] since the metabolic activation process leads to the formation of DNA adducts, which are carcinogen metabolites bound covalently to DNA, usually at guanine or adenine. If DNA adducts escape cellular repair mechanisms and persist, they may lead to miscoding, resulting in a permanent mutation. Using a linear regression model we elucidated whether the increase in 8-OHdG levels in cancer patients is related to the increase in residual damage by hOGG1 FLARE among smokers. A significant correlation, (P < 0.02) was found between high levels of 8-OHdG levels at baseline and high levels of residual damage.

Lung cancer is a logical disease for evaluating oxidative damage and the role of free radicals because the etiologic agents for lung cancer are tobacco carcinogens that are known to damage DNA. We therefore used H_2_O_2 _as the ROS-inducing agent in lymphocytes of lung cancer patients and controls in order to measure differential sensitivity (reflected by level of induced DNA damage) and variation in repair capacity. H_2_O_2 _undergoes a Fe (II)-mediated Fenton reaction in the cell, resulting in the formation of the highly reactive hydroxyl radical and other reactive oxygen species which can induce strand breaks in the DNA [37]. Several studies have shown that the repair of single-strand breaks in normal cells is generally rapid, occurring within one hour from exposure [38,39] which is in agreement with results from our DNA repair time course (Additional file 1, Figure S1B). In our experiments, treatment of the cells with H_2_O_2 _followed by a one-hour incubation (to allow strand breaks repair) showed that the level of induced DNA damage was significantly different in all four groups studied, with 3.36-and 1.76-fold increases in detected DNA damage in the cancer cases and current smoker controls respectively as compared to former smoker controls. These results indicate that the peripheral lymphocytes from the three comparison groups may have differential sensitivity to H_2_O_2 _with the lung cancer patients being more sensitive than the current and former smoker controls.

In addition to the increase in sensitivity to H_2_O_2_-induced damage, the lung cancer cases may have a poorer repair capacity than either group of current smoker controls. Several case-control studies have reported that low DNA repair capacity is an independent risk factor for several types of cancers [27,40,41]. Likewise, several studies have shown similar increases in DNA damage and decreases in repair efficiency in response to in vitro treatment with chemical mutagens [42-44]. In the current study, H_2_O_2 _treatment followed by a one-hour incubation with hOGG1 enzyme allowed the quantification of the level of residual 8-oxoG in DNA and determination of the repair capacity in the two groups. Our results showed that current smoker controls had a significantly lower level of residual DNA damage compared to the cases. After adjustment for age, gender and family history of a smoking-related cancer using linear regression there was a 5-fold increase in risk of lung cancer associated with high levels of residual damage/reduced repair capacity (OR = 5.07; 97% CL = 1.80-14.24). Reduced OGG1 activity could be expected to be a risk factor in other smoking-related cancers. However, given the abundance of 8-oxoguanine and the suspected role of oxidative stress in cancer, reduced OGG1 activity might be associated with the risk of some other cancers as well. The excision of 8-oxoguanine residues by OGG1 protects against aberrant adenine-cytosine and guanine-thymine conversions [15] that can lead to heritable mutagenesis, particularly in non-proliferative cells in which lesions accumulate by cell division [45]. OGG1 expression therefore preserves genomic integrity, and it has been shown that Ogg1-deficient mice experience enhanced incidences of mutations and tumor formation [46].

Furthermore, our results showed that the current smoker controls had a relatively wide range of repair capacity (Figures 1A and 1B) as compared to the cases, the majority of which fell in the poor repair category. Reduced repair capacity of a particular repair protein may only be revealed when expressed in adverse cellular conditions under oxidative stress. The major risk factor for lung cancer is tobacco smoke and a suboptimal DNA repair could play a crucial role in the susceptibility of the disease. Using the FLARE assay, we were able to identify the small percentage of current smoker controls with poor repair who would be good candidates for intensive follow-up and screening.

## Conclusion

In summary, results from this study suggest that high levels of 8-OHdG are correlated with high levels of oxidative DNA residual damage and suboptimal OGG1 repair capacity all of which were predominantly seen in the lung cancer case group. A limitation of our study is the relatively small sample size of the study groups. However, given the striking differences in damage and repair capacities between groups, it is unlikely that the effect is due to the sample size. Large prospective studies are therefore warranted in which robust functional assays such as the ones used in our study could contribute to the identification of individuals. Such an approach, which may be extended to include additional DNA repair pathways, may provide an effective strategy for the prevention of tobacco-related cancers. The simplicity, rapidity and sensitivity of the FLARE assay make it a valuable tool for screening and possibly for prioritizing potential cases for intensive surveillance.

## Competing interests

The authors declare that they have no competing interests.

Financial competing interests:

1. In the past five years have you received reimbursements, fees, funding, or salary from an organization that may in any way gain or lose financially from the publication of this manuscript, either now or in the future? Is such an organization financing this manuscript (including the article-processing charge)? If so, please specify.

Response: No

2. Do you hold any stocks or shares in an organization that may in any way gain or lose financially from the publication of this manuscript, either now or in the future? If so, please specify.

Response: No

3. Do you hold or are you currently applying for any patents relating to the content of the manuscript? Have you received reimbursements, fees, funding, or salary from an organization that holds or has applied for patents relating to the content of the manuscript? If so, please specify.

Response: No

4. Do you have any other financial competing interests? If so, please specify. Non-financial competing interests: are there any non-financial competing interests (political, personal, religious, academic, ideological, intellectual, commercial or any other) to declare in relation to this manuscript? If so, please specify.

Response: No

## Authors' contributions

REZ was responsible for conceiving and designing the study as well as analyzing and interpreting the data and writing the different stages of the manuscript. CMM was responsible for the Comet/FLARE experiments and contributed in the data analysis and drafting the manuscript. AC was responsible for the ELISA assays and contributed in the data analysis. MRS and AG were responsible for coordination of the subject recruitment for the study and participated in data interpretation and manuscript preparation. CEJ was responsible for data analysis and contributed to manuscript preparation. All authors read and approved the final manuscript.

## Pre-publication history

The pre-publication history for this paper can be accessed here:

http://www.biomedcentral.com/1471-2407/10/439/prepub

## Supplementary Material

Additional file 1 **Supplementary results**. Figure S1: A) Mean cell viability in response to different concentrations of H_2_O_2_. B) Repair over time in a normal cell-line after 60 μM treatment with H_2_O_2_. Click here for file

## References

[B1] McHugh PJ Spanswick VJ Hartley JA Repair of DNA interstrand crosslinks: molecular mechanisms and clinical relevance Lancet Oncol 2001 2 8 483 90 10.1016/S1470-2045(01)00454-5 11905724

[B2] Berwick M Vineis P Markers of DNA repair and susceptibility to cancer in humans: an epidemiologic review J Natl Cancer Inst 2000 7; 92 11 874 97 10.1093/jnci/92.11.874 10841823

[B3] Loft S Paulsen HE Cancer risk and oxidative DNA damage in man J Mol Med 1996 74 297 312 10.1007/BF00207507 8862511

[B4] Bjelland S Seeberg E Mutagenicity, toxicity and repair of DNA base damage induced by oxidation Mutat Res 2003 531 37 80 14637246 10.1016/j.mrfmmm.2003.07.002

[B5] Caporaso N The molecular epidemiology of oxidative damage to DNA and cancer J Natl Cancer Inst 2003 95 1263 1265 12953074 10.1093/jnci/djg065

[B6] Cooke MS Evans MD Dizdaroglu M Lunec J Oxidative DNA damage: mechanisms, mutation, and disease FASEB J 2003 17 10 1195 214 10.1096/fj.02-0752rev 12832285

[B7] Hayes JD McLellan LI Glutathione and glutathione-dependent enzymes represent a co-ordinately regulated defence against oxidative stress Free Radic Res 1999 31 4 273 300 10.1080/10715769900300851 10517533

[B8] Hecht SS Tobacco smoke carcinogens and lung cancer J Natl Cancer Inst 1999 91 1194 10 10.1093/jnci/91.14.1194 10413421

[B9] Spitz MR Wei Q Dong Q Amos CI Wu X Genetic susceptibility to lung cancer: the role of DNA damage and repair Cancer Epidemiol Biomarkers Prev 2003 12 8 689 98 12917198

[B10] Deng L Kimmel M Foy M Spitz M Wei Q Gorlova O Estimation of the effects of smoking and DNA repair capacity on coefficients of a carcinogenesis model for lung cancer Int J Cancer 2009 124 9 2152 8 10.1002/ijc.24149 19123470 PMC2749693

[B11] Smart DJ Chipman JK Hodges NJ Activity of OGG1 variants in the repair of pro-oxidant-induced 8-oxo-2'-deoxyguanosine DNA Repair [Amst] 2006 5 11 1337 45 10.1016/j.dnarep.2006.06.001 16861056

[B12] Dizdaroglu M Substrate specificities and excision kinetics of DNA glycosylases involved in base-excision repair of oxidative DNA damage Mutat Res 2003 531 1-2 109 26 14637249 10.1016/j.mrfmmm.2003.07.003

[B13] Nakabeppu Y Regulation of intracellular localization of human MTH1, OGG1, and MYH proteins for repair of oxidative DNA damage Prog Nucleic Acid Res 2001 68 75 94 Mol Biolfull_text 10.1016/s0079-6603(01)68091-7 11554314

[B14] Kasai H Analysis of a form of oxidative DNA damage, 8-hydroxy-2'-deoxyguanosine, as a marker of cellular oxidative stress during carcinogenesis Mutat Res 1997 387 3 147 63 10.1016/S1383-5742(97)00035-5 9439711

[B15] Moriya M Single-stranded shuttle phagemid for mutagenesis studies in mammalian cells: 8-oxoguanine in DNA induced targeted G·C → T·A transversions in simian kidney cells Proc Natl Acad Sci USA 1993 90 1122 6 10.1073/pnas.90.3.1122 8430083 PMC45823

[B16] Valko M Izakovic M Mazur M Rhodes CI Telser J Role of oxygen radicals in DNA damage and cancer incidence Mol Cell Biochem 2004 266 1-2 37 56 10.1023/B:MCBI.0000049134.69131.89 15646026

[B17] Bartsch H DNA adducts in human carcinogenesis: Etiological relevance and structure-activity relationship Mut Res Rev Genet Toxicol 1996 340 67 79 10.1016/s0165-1110(96)90040-8 8692183

[B18] Hansen WK Kelley MR Review of mammalian DNA repair and translational implications J Pharmacol Exp Ther 2000 295 1 9 10991953

[B19] Minowa O Masanori Hirano T Monden Y Nakai S Fukuda M Itoh M Takano H Hippou Y Aburatani H Masumura K Mmh/Ogg1 gene inactivation results in accumulation of 8-hydroxyguanine in mice Proc Natl Acad Sci USA 2000 97 4156 4161 10.1073/pnas.050404497 10725358 PMC18180

[B20] Collins AR Ai-guo M Duthie SJ The kinetics of repair of oxidative DNA damage [strand breaks and oxidised pyrimidines] in human cells Mutat Res 1995 36 1 69 77 10.1016/0921-8777(94)00043-6 7528897

[B21] Kamiya H Yamaguchi A Suzuki T Harashima H Roles of specialized DNA polymerases in mutagenesis by 8-hydroxguanine in human cells Mutat Res 2010 686 1-2 90 95 20153758 10.1016/j.mrfmmm.2010.02.001

[B22] Shen J Deininger P Hunt JD Zhao H 8-Hydroxy-2'-deoxyguanosine [8-OH-dG] as a potential survival biomarker in patients with nonsmall-cell lung cancer Cancer 2007 109 3 574 80 10.1002/cncr.22417 17154177

[B23] Paz-Elizur T Krupsky M Blumenstein S Elinger D Schechtman E Livneh Z DNA repair activity for oxidative damage and risk of lung cancer J Natl Cancer Inst 2003 95 17 1312 9 12953085 10.1093/jnci/djg033

[B24] Fairbairn DW Olive PL O'Neill KL The comet assay: a comprehensive review Mutat Res 1995 339 1 37 59 7877644 10.1016/0165-1110(94)00013-3

[B25] Singh NP McCoy MT Tice RR Schneider EL A simple technique for quantitation of low levels of DNA damage in individual cells Exp Cell Res 1988 175 1 184 91 10.1016/0014-4827(88)90265-0 3345800

[B26] Gaivão I Piasek A Brevik A Shaposhnikov S Collins AR Comet assay-based methods for measuring DNA repair in vitro; estimates of inter- and intra-individual variation Cell Biol Toxicol 2009 25 1 45 52 10.1007/s10565-007-9047-5 18058031

[B27] Marcon F Andreoli C Rossi S Verdina A Galati R Crebelli R Assessment of individual sensitivity to ionizing radiation and DNA repair efficiency in a healthy population Mutat Res 2003 541 1-2 1 8 14568289 10.1016/s1383-5718(03)00171-2

[B28] Rosenquist TA Zharkov DO Grollman AP Cloning and characterization of a mammalian 8-oxoguanine DNA glycosylase Proc Natl Acad Sci USA 1997 94 7429 7434 10.1073/pnas.94.14.7429 9207108 PMC23838

[B29] Smith CC O'Donovan MR Martin EA hOGG1 recognizes oxidative damage using the comet assay with greater specificity than FPG or ENDOIII Mutagenesis 2006 21 3 185 90 10.1093/mutage/gel019 16597659

[B30] Udumudi A Jaiswal M Rajeswari N Desai N Jain S Balakrishna N Rao KV Ahuja YR Risk assessment in cervical dysplasia patients by single cell gel electrophoresis assay: a study of DNA damage and repair Mutat Res 1998 412 2 195 205 9539974 10.1016/s1383-5718(97)00164-2

[B31] Blasiak J Arabski M Krupa R Wozniak K Rykala J Kolacinska A Morawiec Z Drzewoski J Zadrozny M Basal, oxidative and alkylative DNA damage, DNA repair efficacy and mutagen sensitivity in breast cancer Mutat Res 2004 554 1-2 139 48 15450412 10.1016/j.mrfmmm.2004.04.001

[B32] Vodicka P Polivkova Z Sytarova S Demova H Kucerova M Vodickova L Polakova V Naccarati A Smerhovsky Z Ambrus M Cerna M Hemminki K Chromosomal damage in peripheral blood lymphocytes of newly diagnosed cancer patients and healthy controls Carcinogenesis 2010 in press 10.1093/carcin/bgq056 20215138

[B33] Wiencke JK Thurston SW Kelsey KT Varkonyi A Wain JC Mark EJ Christiani DC Early age at smoking initiation and tobacco carcinogen DNA damage in the lung J Natl Cancer Inst 1999 91 7 614 9 10.1093/jnci/91.7.614 10203280

[B34] Chen L Wang M Villalta PW Luo X Feuer R Jensen J Hatsukami DK Hecht SS Quantitation of an acetaldehyde adducts in human leukocyte DNA and the effect of smoking cessation Chem Res Toxicol 2007 20 1 108 13 10.1021/tx060232x 17226933 PMC2518843

[B35] Mizoue T Kasai H Kubo T Tokunaga S Leanness, smoking, and enhanced oxidative DNA damage Cancer Epidemiol Biomarkers Prev 2006 15 3 582 5 10.1158/1055-9965.EPI-05-0658 16537720

[B36] Hatt L Loft S Risom L Møller P Sørensen M Raaschou-Nielsen O Overvad K Tjønneland A Vogel U OGG1 expression and OGG1 Ser326Cys polymorphism and risk of lung cancer in a prospective study Mutat Res 2008 639 1-2 45 54 18155253 10.1016/j.mrfmmm.2007.11.002

[B37] loyd DR Phillips DH Oxidative DNA damage mediated by copper[II], iron[II] and nickel[II] fenton reactions: evidence for site-specific mechanisms in the formation of double-strand breaks, 8-hydroxydeoxyguanosine and putative intrastrand cross-links Mutat Res 1999 424 1-2 23 36 10064847 10.1016/s0027-5107(99)00005-6

[B38] Collins AR Dusinská M Oxidation of cellular DNA measured with the comet assay Methods Mol Biol 2002 186 147 59 12013763 10.1385/1-59259-173-6:147

[B39] Torbergsen AC Collins AR Recovery of human lymphocytes from oxidative DNA damage; the apparent enhancement of DNA repair by carotenoids is probably simply an antioxidant effect Eur J Nutr 2000 39 2 80 5 10.1007/s003940050006 10918989

[B40] Hjertvik M Erixon K Ahnström G Repair of DNA damage in mammalian cells after treatment with UV and dimethyl sulphate: discrimination between nucleotide and base excision repair by their temperature dependence Mutat Res 1998 407 2 87 96 9637237 10.1016/s0921-8777(97)00062-1

[B41] Zheng YL Loffredo CA Yu Z Jones RT Krasna MJ Alberg AJ Yung R Perlmutter D Enewold L Harris CC Shields PG Bleomycin-induced chromosome breaks as a risk marker for lung cancer: a case-control study with population and hospital controls Carcinogenesis 2003 24 2 269 74 10.1093/carcin/24.2.269 12584177

[B42] Schmid O Speit G Genotoxic effects induced by formaldehyde in human blood and implications for the interpretation of biomonitoring studies Mutagenesis 2007 22 1 69 74 10.1093/mutage/gel053 17158519

[B43] Speit G Hartmann A The comet assay: a sensitive genotoxicity test for the detection of DNA damage and repair Methods Mol Biol 2006 314 275 86 full_text 16673888 10.1385/1-59259-973-7:275

[B44] Lacoste S Castonguay A Drouin R Repair kinetics of specific types of nitroso-induced DNA damage using the comet assay in human cells Mutat Res 2007 624 1-2 18 30 17612576 10.1016/j.mrfmmm.2007.02.030

[B45] Oka S Ohno M Tsuchimoto D Sakumi K Furuichi M Nakabeppu Y Two distinct pathways of cell death triggered by oxidative damage to nuclear and mitochondrial DNAs EMBO J 2008 27 2 421 32 10.1038/sj.emboj.7601975 18188152 PMC2234344

[B46] Klungland A Rosewell I Hollenbach S Larsen E Daly G Epe B Seeberg E Lindahl T Barnes DE Accumulation of premutagenic DNA lesions in mice defective in removal of oxidative base damage Proc Natl Acad Sci USA 1999 96 23 13300 5 10.1073/pnas.96.23.13300 10557315 PMC23942

