# Supplementary material for: Rapid method for determination of DNA repair capacity in human peripheral blood lymphocytes amongst smokers
Source: BMC Cancer. 2010 Aug 18;10:439. doi: 10.1186/1471-2407-10-439 (PMC2933626; doi:10.1186/1471-2407-10-439)
Supplement: Additional file 1 — Supplementary results. Figure S1: A) Mean cell viability in response to different concentrations of H2O2. B) Repair over time in a normal cell-line after 60 μM treatment with H2O2. [file 1471-2407-10-439-S1.DOC]

**Supplementary results.**

Figure S1 **A)** Mean cell viability in response to different concentrations of H_2_O_2_. **B)** Repair over time in a normal cell-line after 60μM treatment with H_2_O_2_.

**A**

**B**
